# Supplementary material for: Early life predictors of late life cerebral small vessel disease in four prospective cohort studies
Source: Brain. 2021 Sep 28;144(12):3769–78. doi: 10.1093/brain/awab331 (PMC8719837; doi:10.1093/brain/awab331)
Supplement: awab331_Supplementary_Data [file awab331_supplementary_data.pdf]

Supplementary materials

Supplementary figure 1: Recruitment flow chart of STRADL (A), the Dutch Famine Birth Cohort (B), LBC 1936 (C) and the Simpson's cohort (D).

A. STRADL

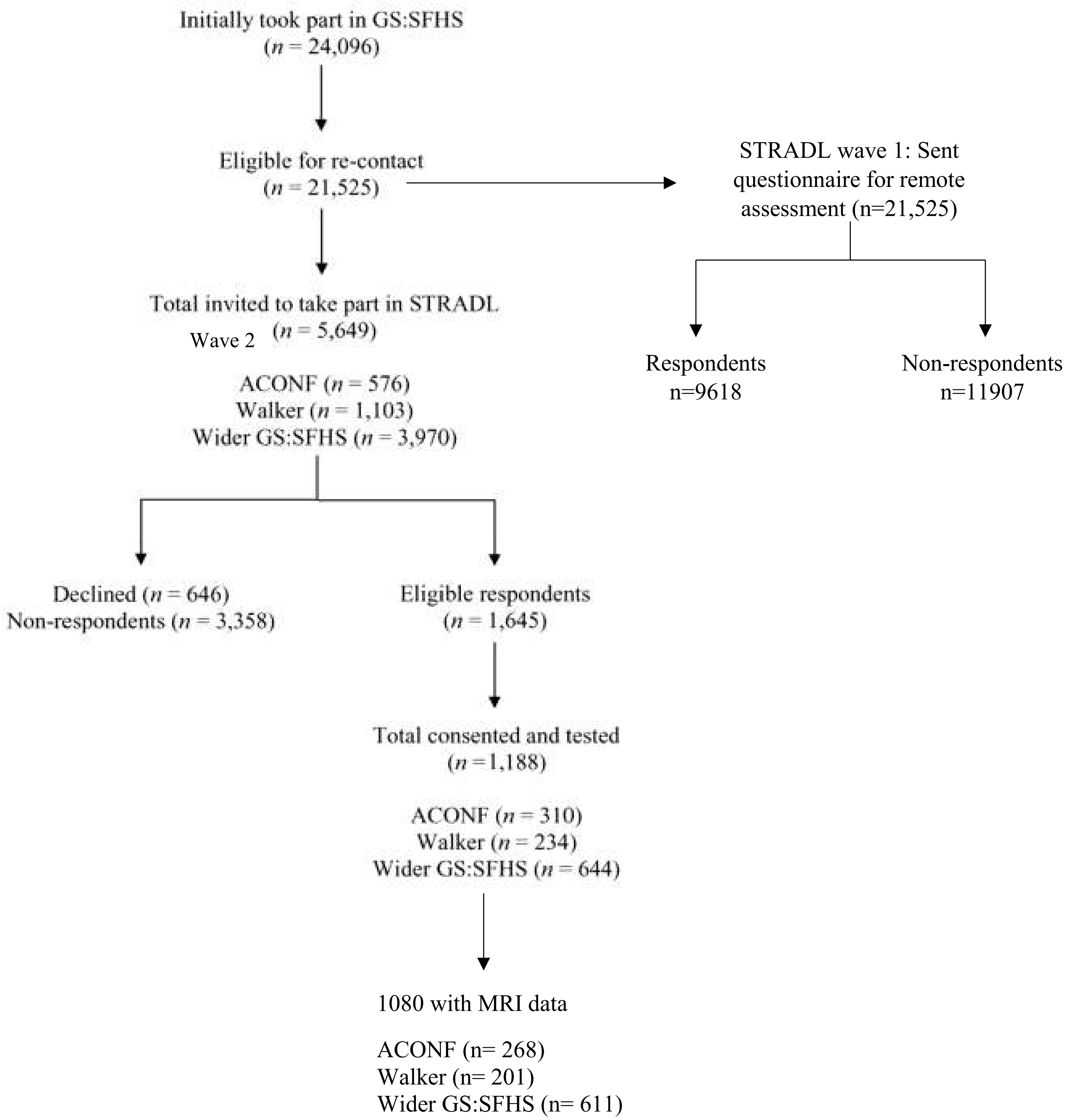

GS:SFHS= Generation Scotland: Scottish Family Health Study

ACONF= Aberdeen Children of the 1950's cohort.

Adapted from: Habota T, Sandu AL, Waiter GD et al. Cohort profile for the STratifying Resilience and Depression Longitudinally (STRADL) study: A depression-focused investigation of Generation Scotland, using detailed clinical, cognitive, and neuroimaging assessments [version 1]. Wellcome Open Res 2019, 4:185 (doi: 10·12688/wellcomeopenres.15538·1)

B. The Dutch Famine Birth Cohort

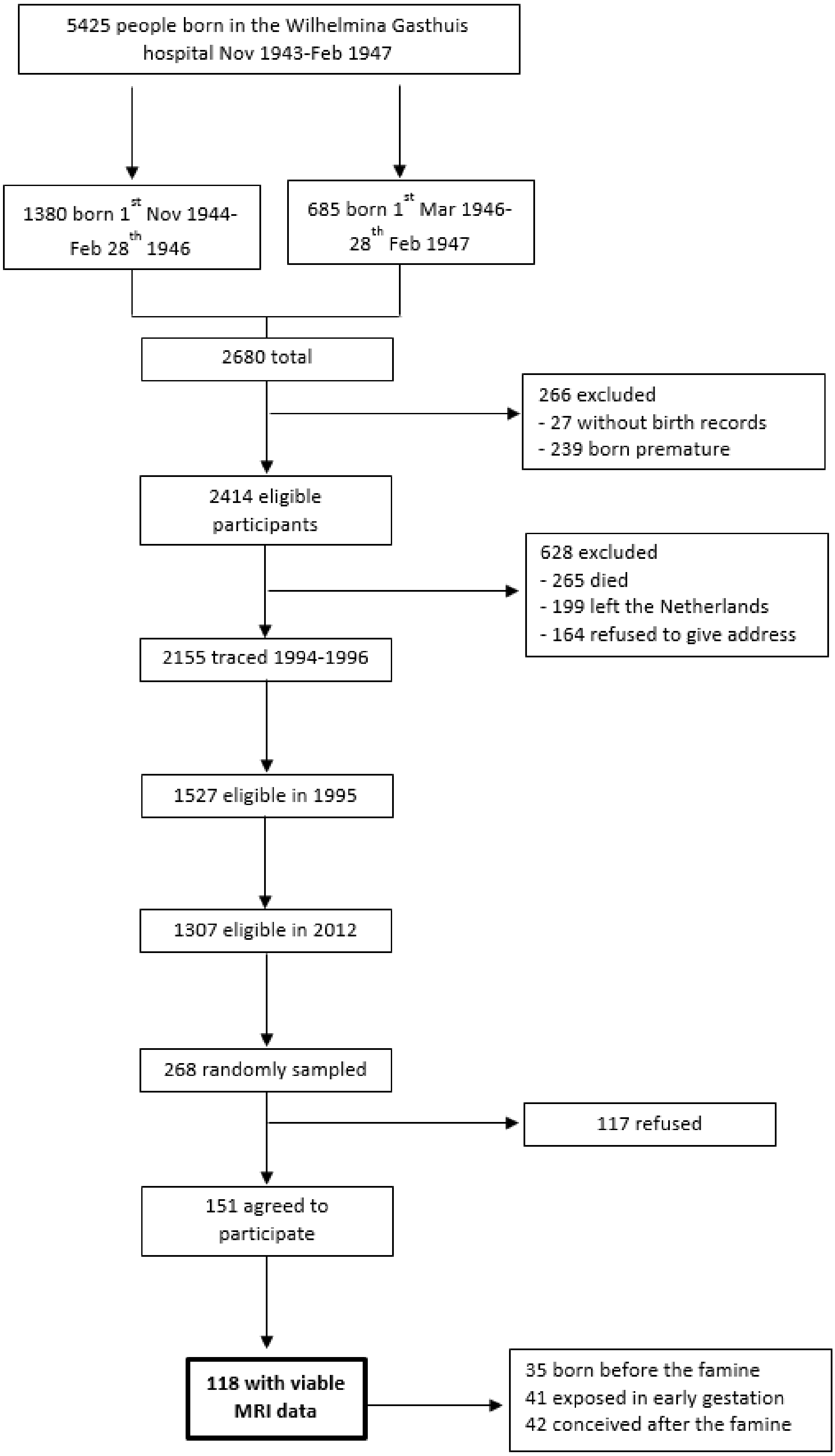

C. The Lothian Birth Cohort 1936

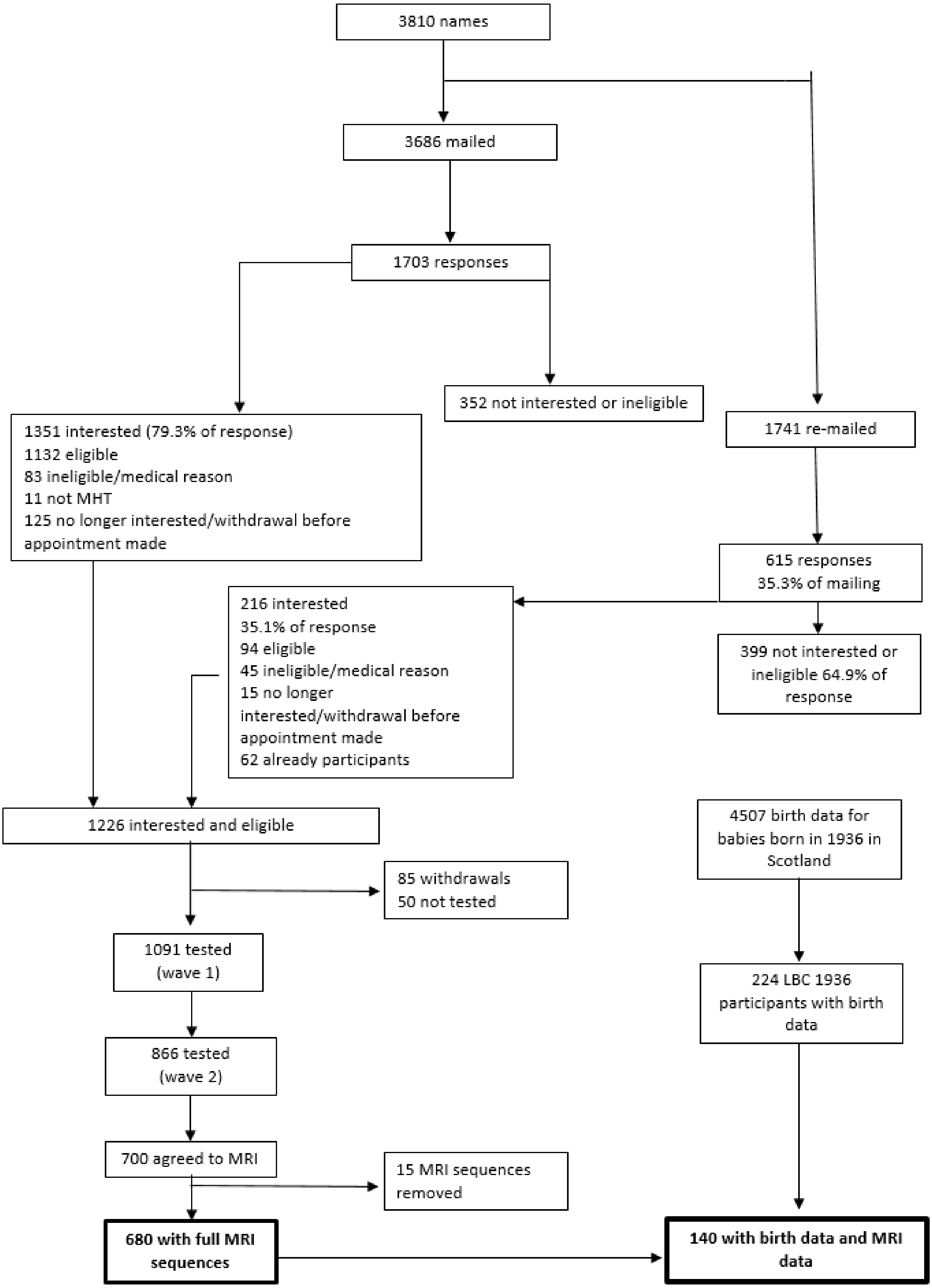

Adapted from: Deary, BMC Geriatrics 2007, 7:28

D. The Simpson's Birth Cohort Study

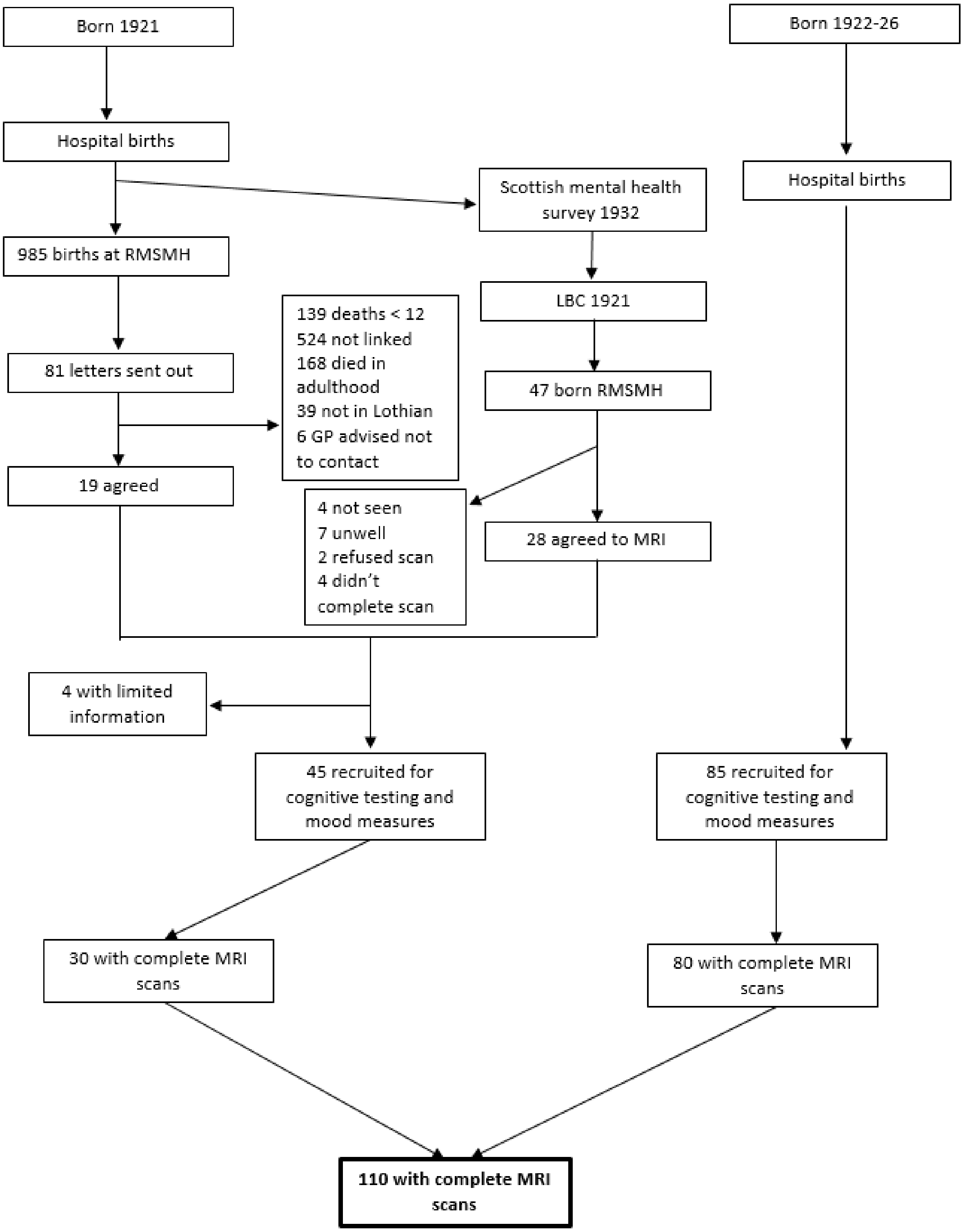

RMSM= Royal Maternity and Simpson Memorial hospital

Adapted from: Shenkin, SD (2006) *Life course influences on cognitive ability and cerebrovascular disease* (MD thesis)

Supplementary table 1: Details of early life factors available in all cohorts

Early life factor	STRADL	Dutch Famine Birth Cohort	LBC 1936	Simpson's cohort	Descriptive statistics
Birth weight	**Available for:** 1) all ACONF participants 2) 154 Walker participants.<br>**Taken from**: 1). the ACONF database which had birth records taken from the Aberdeen Maternity and Neonatal database at the Aberdeen Maternity Hospital. Recorded in pounds and ounces and converted to grams.<br>2·) the Walker database which had birth records in Dundee. Recorded in grams.	**Available for:** all participants<br>**Taken from**: birth records at the Wilhelmina Gasthuis Hospital in Amsterdam. Recorded in grams	**Available for:** 140 participants<br>**Taken from**: birth records from the Simpson Memorial Hospital, Bellshill Hospital Lanarkshire and Aberdeen Maternity Hospital. Recorded in pounds and ounces and converted to grams.	**Available for:** all participants<br>**Taken from**: birth records from the Royal Maternity and Simpson Hospital, the Elsie Memorial Hospital and the Lyin-in Institution. Recorded in pounds and ounces and converted to grams	Mean (SD)<br>**STRADL:** 2·4% participants with birth weight <5lbs; 3309·3 (SD 529·4)<br>**Dutch Famine Birth Cohort**: 3417·5g (SD 503·4)<br>**LBC 1936:** 3351·5g (SD 482·1)<br>**Simpson's cohort:** 3333·6g (SD 457·2);<br><br>**ANOVA:** F(3, 365)= 0·99, p=0·37
Ponderal index	Not collected	**Available for:** all participants<br>**Calculated from**: birth weight and length as birth weight/birth length^3	**Available for:** 79 participants<br>**Calculated from**: birth weight and length birth weight/birth length^3	**Available for:** all participants<br>**Calculated from**: birth weight and length birth weight/birth length^3	Mean (SD)<br>**Dutch Famine cohort:** 26·16 (SD 2·30)<br>**LBC 1936:** 27·33 (SD 5·29)<br>**Simpson's cohort:** 25·55 (SD 4·10)<br><br>**ANOVA:** F(2, 297)= 4·72, p=0·01
Childhood IQ	**Available for:** all ACONF participants<br>**Calculated by:** adjusting the raw scores from the Schonnell and Adams Essential Intelligence Test (age 9 IQ), for age in days at testing and placed on an IQ type scale (mean=100, SD=15).	Not available	**Available for**: all participants<br>**Calculated by:** adjusting the raw scores from the Moray House Test number 12 (MHT) (age 11 IQ), for age in days at testing and placed on an IQ type scale (mean=100, SD=15).	**Available for:** 47 participants<br>**Calculated by:** adjusting the raw scores from the Moray House Test number 12 (MHT) (age 11 IQ), for age in days at testing and placed on an IQ type scale (mean=100, SD=15).	Mean (SD)<br>**STRADL:** 102·0 (8·9)<br>**LBC 1936:** 100·80 (SD 15·3)<br>**Simpson's cohort:** 101·7 (14·5)<br><br>**ANOVA:** (F(2)= 1·35, p= 0·26
Education	**Available for:** all participants<br>**Measured:** divided into compulsary, more than compulaary and post secondary and dichotomised at compulsary.	**Available for:** all participants<br>**Measured:** a 10 point scale (1= Primary education not completed, 10=University completed) and dichotomised at lower secondary school	**Available for:** all participants<br>**Measured:** using years of education and dichotomised at 11 years.	**Available for:** all participants<br>**Measured:** using years of education and dichotomised at 11 years.	% of participants with low education (≤ 11 years)<br><br>**STRADL** 24%; **Dutch Famine** 62·7%<br>**LBC 1936** 71·7%<br>**Simpson's cohort** 80·9%<br><br>**Chi square:**<br>□ ^{2}(3)= 8·0, p=0·05
Childhood SES	**Available for:** all participants<br>**Obtained from:** self-reports by each participant at the time of the interview in adulthood and classified as manual and non-manual	**Available for:** all participants<br>**Obtained from**: birth records We classified childhood SES as manual and non-manual at the time of the child's birth	**Available for:** all participants<br>**Obtained from:** self-reports by each participant at the time of the interview in adulthood and checked using the General Register Office's Census, 1951. Classification of Occupations We classified childhood	**Available for:** all participants<br>**Obtained from:** self-reports by each participant at the time of the interview in adulthood and classified as manual and non-manual at age 11	% of participants with low childhood SES (manual parental occupation)<br><br>**STRADL:** 67·2%<br>**Dutch famine birth cohort:** 66·7%<br>**LBC 1936:** 74·2%<br>**Simpson's cohort:** 69·1%

			SES as manual and non-manual at age 11		**Chi square:** □^{2}(3)= 5·7, p=0·13

Supplementary table 2: Sequence parameters for MRI scanning in all cohorts

Sequence name	Acquisition method	Field of view (mm)	Matrix	Slices	Thickness (mm)	Voxel (mm)	TR/TE/TI (ms)
**STRADL (Aberdeen)**							
T2-weighted	3D TSE	250	512 x 512	320	0·5	0·5 x 0·5 x 0·5	2500/314
SWI	FFE	230	768 x 768	130	1	0·3 x 0·3 x 1.0	31/7·2,14·4,20·6,26·8
FLAIR	3D IR FSE	240	256 x 256	160	1	0·94 x 0·94 x 1.0	8000/349/2400
T1-weighted volume	3D IR TFE	240	256	160	1	1.0 x 1.0 x 1.0	8·3/3·8/1031
**STRADL (Dundee)**							
T2-weighted	3D TSE	256	256 x 256	320	0·5	0·5 x 0·5 x 0·5	3200/ 408
SWI	FFE	230	320 X 160	144	1.0	0·4 x 0·4 x 1.0	28/20
FLAIR	3D IR FSE	256	256 X 256	160	1.0	1.0 x 1.0 x 1.0	5000/386/1800
T1-weighted volume	3D IR TFE	256	256 x256	208	1.0	1.0 x 1.0 x 1.0	1740/ 2·62/ 900
**Dutch Famine Birth cohort**							
T2-weighted	2D TSE	230 x 230	512 x 512	28	4	0·45 x 0·45	3000/80
SWI-weighted	3D SWI	220 x 220	448 x 448	220	1.2	0·49 x 0·49 x 0·6	19·8/25·7
FLAIR	3D	250 x 250	240 x 240	321	1.1	1.1 x 1.1 x 1.12	4800/365
T1-weighted volume	3D TFE	256 x 256	256 x 256	180	1.0	1.1 x 1.1 x 1.2	6·8/3·1
**LBC 1936**							
T2-weighted	FSE	256 x 256	256 x 256	80	2	1 x 1 x 2	1 1320/105
T2*-weighted	Gradient echo	256 x 256	256 x 192*	80	2	1 x 1 x 2	940/15
FLAIR	FSE	256 x 256	256 x 192*	40	4	1 x 1 x 4	9002/147/2200
T-1 weighted mapping	FSPGR	256 x 256	128 x 128*	72	2	1 x 1 x 2	6/2
T1-weighted volume	3D IR-Prep FSPGR	256 x 256	192 x 192*	160	1.3	1 x 1 x 1.3	10/4/500
**Simpson's cohort**							
T2-weighted	FSE	240 x 240	256 x 256	-	5	-	6300/102
T2*-weighted	Gradient echo	-	-	-	-	-	-

FLAIR	FSE	240 x 240	256 x 192	-	5	-	9000/140/2200
T-1 weighted mapping	FSPGR	240 x 240	256 x 224	-	5	-	450/8
T1-weighted volume	3D IR-Prep FSPGR	240 x 240	256 x 256	-	1.7	-	400

* Zero filled to 256 x 256; TR: repetition time; TE: echo time; FLAIR: Fluid-attenuated inversion recovery; SWI: susceptibility weighted imaging; IR-Prep: inversion recovery prepared; FSPGR: fast spoiled gradient echo; FSE: fast spin echo. – is used where data were not availabl

Comparison of participants with and without MRI.

Those who declined MRI were older (STRADL U= 54087.5, p=0.01; Dutch Famine Birth cohort (U= 1353.5, p=001); Simpson cohort (U=530.5, p<0.001) and more likely to be diagnosed hypertensive (Simpson cohort χ^2 (1)= 4.4, p=0.036) and diabetic (STRADL only χ^2(1)= 14.4, p<0.001). In STRADL and the LBC1936 they were also more likely to have lower childhood IQ (STRADL U= 1947.5, p=0.03; LBC1936 U= 110284.0, p=0.01) and lower adult and childhood social class (STRADL only; manual vs non-manual adult occupation: χ^2 (1)=9.2, p<0.01), manual vs non-manual Father's occupation χ^2 (1)= 4.5, p=0.04).

Comparison of participants and non-participants.

Participants in this wave of the Dutch Famine Birth cohort were randomly sampled from 1307 eligible cohort members. Participants in this study had a higher birth weight than eligible non-participants (3438g vs 3346g). Data were not available to compare additional characteristics between participants and eligible non-participants. In STRADL those who participated in the face to face assessment in this study were older (median age 62) with higher levels of education (40% with university education) compared to the previous wave of STRADL and the GS:SFHS population. In the previous wave of STRADL respondents to the remote assessment were predominantly female (62%) and were older (mean 50.48 SD 13.41) and from less socioeconomically deprived areas compared with non-respondents in the Scottish Index of Multiple Deprivation (SIMD) 2009 (4123 vs 3733)[15]. Participants in this this wave of the LBC1936 were less likely to be hypertensive, have high cholesterol and smoke at wave 1 than those who declined to participate and less likely to have low education and childhood IQ.

Supplementary Table 3: Key characteristics of STRADL face-to-face assessment broken down by MRI status, STRADL remote follow up, wider GS:SFHS baseline assessment and the wider Scottish population.

			**STRADL**				
	Scottish Population[59,60]	GS:SFHS total (N=21525)[61]	Wave 1 Remote assessment		**This study Face-to face assessment (n= 1,188)**		
			Non-respondents (N=11907)	Respondents (N= 9618)	All (n= 1,188)	With MRI (n=1076)	Without MRI (n=109)
Gender (n, % female)	51.5	41	57	62§	59	436 (40.5)	55 (50.5)
Mean age	-	-	-	-	59.3 (10.1)	59.3 (10.2)	62.1(8.7)*
Median age Male	-	47	43	54	62	62	62
Median age Female	-	48	45	52	61	61	61
Hypertension, %	66	27.2	-	-	299 (27.7)	299 (27.7)	40 (33.6)
Diabetes, %	5	6.9	-	-	84 (7.9)	69 (6.4)	19 (16.0)**
Current smokers %	21	35	-	-	435 (40.3)	435 (45.3)	45 (44.6)
Annual income >£30,000 (%)		60	57	63	-	-	-
SIMD		3910 (1842)	3733 (1875)	4123 (1777)	-	-	-
Adult SES, manual	-	-	-	-	345 (31.9)	344 (32.2)	52 (46.4)**
Employment in those aged up to 75 years (%)							
Unemployed	5	1.7	5	4	3	-	-
Retired	12.9	15.1	13	18	32	-	-
Employed	69	62.8	71	71	65	-	-
Education (%)							
Degree	26	33	28	37	40	-	-
No qualifications	27	5	9	7	25	-	-
Childhood IQ, mean (SD)	-	-	-	-	102.0 (8.9)	102.5 (8.8)	97.4 (12)*
Low education, n (%)	-	-	-	-	259 (24.0)	259 (24.0)	33 (29.7)

Manual childhood SES	-	-	-	-	719 (67.2)	718 (67.2)*	84 (77.1)*
Birth weight	-	-	-	-	3309.3 (529.4)	3309.3 (529.4)	3309.3 (436.5)

§ t(21457)= -31.25, p<0.001, cohen's d=0.21)[15]; * those with MRI vs those without MRI p<0.05; ** those with MRI vs those without MRI p<0.01
Abbreviations: SIMD the Scottish Index of Multiple Deprivation 2009: numbers represent mean rank (SD); GS:SFHS Generation Scotland Scottish Family Health Study; SES socioeconomic status; - Is used where data are not available.

Supplementary table 4: Key characteristics of the Dutch Famine Birth cohort in the current study broken down by MRI status, previous waves and the wider Dutch population.

			1994-1996 (Aged 50)[66]	2002-2004 (Aged 58)[67]	This study 2012-2013 (Aged 68)		
	Dutch population[62,63,64,65]	Original cohort (n=2414)[66]	(n=741)	(n=721)	All participants (n=151)	With MRI (n=118)	Without MRI (n=33)
Age, mean (SD)					67.5 (0.9)	67.5** (0.9)	68.0** (0.8)
Females, %	50.5	48.6	52	53	55.6	66 (55.9)	18 (54.5)
Manual occupation (mother), %	-	-	70	74	67.5	44 (37.3)	18 (54.5)
Birth weight (g), mean (SD)	-	3346 (478)	3347	3363 (465)	3438.3* (488.0)	3417.5 (503.4)	3512.4 (427.5)
Ponderal index, mean (SD)	-	26.4 (2.4)	26.3 (2.3)	26.3 (2.3)	26.2 (2.3)	26.2 (2.3)	26.7 (2.6)
Hypertension, %	20.1	-			54.7	62 (53)	20 (60.6)
Aged 50-59	33.7	-					
Systolic BP, mean (SD)		-	126 (16)	137 (18)	151 (18)	152 (17)	148 (23)
Diabetes, %		-			29 (19.2)	24 (20.3)	5 (15.2)
Hypercholesterolimia, %		-			70 (47.3)	56 (47.9)	14 (45)
Smoking, current, %	19	-	34	23	17 (11.3)	13 (11.0)	4 (12.1))
SES (ISEI)	46	-	48 (13)	47 (40-59)	51 (16-77)	51 (16-77)	43 (25-76)
Low education, %	-	-	66		91 (60.3)	74 (62)	17 (52)
Low childhood SES, n (%)		-			83 (67.5)	64 (66.7)	19 (70.4)

* Participants in this study vs eligible non-participants p<0.05; Abbreviations: SES socioeconomic status; ISEI A standard international socio-economic index of occupational status (higher number represents higher socioeconomic status).

Supplementary table 5: Key characteristics of wave 1, wave 2 and those who refused wave 2 of the LBC1936 compared to the wider Scottish population. Characteristics of participants in wave 2 are further broken down by MRI status.

		Wave 1		This study (Wave 2)		
	Scottish population[60,68]	Wave 1 all (n=1091)	Refused wave 2 (n=225)	All (n=866)	MRI only (n=685)	Without MRI (n=186)
Median age	-	69.5 (0.8)	69.6 (0.8)	72.7 (0.7)	72.7 (0.7)	72.8 (0.7)
Gender (n, % female)	51.5	543 (49.8)	125 (55.6)	418 (48.3)	359 (52.8)	89 (47.8)
Adult SES¥	-	2.4 (0.91)	2.6 (0.86)	2.4 (0.9)	2.4 (0.9)	2.4 (0.9)
Adult SES, manual, n (%)	-	232 (21.7)	56 (25.5)	176 (20.7)	144 (79.6)	37 (20.4)
Current smokers, n (%)	31	125 (11.5)	49 (21.8)**	73 (8.4)	56 (8.2)	17 (9.1)
Hypertension, n (%)	41	433 (39.7)	103 (45.8)*	425 (49.1)	334 (49.1)	91 (48.9)
Diabetes, n (%)	3.8	91 (8.3)	16 (7.1)	95 (11)	70 (10.3)	25 (13.4)
Hypercholesterolemia, n (%)	-	386 (35.4)	93 (41.3)*	293 (33.9)	285 (41.9)	71 (38.2)
Childhood IQ, mean (SD)	-	100.0 (14.9)	97.4 (13.4)**	100.7 (15.3)	100.8 (15.3)	100.1 (0.6)∞
Education (%)						
Degree	20	158 (14.5)	25 (11.1)	133 (15.4)	110 (16.2)	23 (12.4)
No qualifications	33	193 (17.7)	40 (17.8)	153 (17.7)	116 (17.1)	37 (19.9)
Low education n (%)	-	812 (74.4)	184 (81.8)**	628 (72.5)	487 (71.6)	141 (75.8)
Father's SES¥	-	2.91 (0.94)	2.87 (0.94)	2.92 (0.9)	2.9 (0.9)	2.9 (1.1)
Manual childhood SES, n (%)	-	700 (72.9)	121 (53.8)	579 (73.5)	460 (74)	119 (71.7)
Birth weight (g), mean (SD)	-	3299.3 (490.5)	3223.1 (476.8)	3323.2 (493.7)	3348.9 (482.9)	3215.1 (531)
Ponderal index, mean (SD)	-	26.9 (5.0)	25.8 (4.1)	27.3 (5.2)	27.4 (5.3)	27.2 (4.8)

* Those who refused wave 2 vs participants of wave 2 $p<0.05$; **Those who refused wave 2 vs participants of wave 2 $p<0.01$; ∞those with MRI vs those without MRI $p<0.05$; Abbreviations: SES socioeconomic status ; ¥ Father's SES and adult SES are based on five categories from professional (1) to unskilled labour (5) (higher number indicates lower SES; Scottish population figures are given in % only; - is used when data were not available.

Supplementary references.

60. Gray L., Leyland AH., Currie E., Montagu I., Bardsley D., Terje A. et al (2016) Scottish Health Survey 2016, Volume 1- main report. https://www.gov.scot/publications/scottish-health-survey-2016-volume-1-main-report/pages/47/

61. 61 Office for National Statistics. (2020). *2011 Census: Aggregate Data*. [data collection]. UK Data Service. SN: 7427, http://doi.org/10.5257/census/aggregate-2011-2.

62. Smith BH, Campbell A, Linksted P, et al.: Cohort profile: Generation Scotland: Scottish Family Health Study (GS:SFHS). The study, its participants and their potential for genetic research on health and illness. Int J Epidemiol. 2013; 42(3): 689–700.

63. Statistics Netherlands (2014) Dutch Census 2011. Central Bureau of statistics Netherlands. https://www.cbs.nl/en-gb/publication/2014/47/dutch-census-2011

64. Schelleman, H., Klungel, O., Kromhout, D. et al. Prevalence and determinants of undertreatment of hypertension in the Netherlands. J Hum Hypertens 18, 317–324 (2004). https://doi.org/10.1038/sj.jhh.1001672

65. Smoking, alcohol and drugs: Health Survey/Lifestyle Monitor, Statistics Netherlands (CBS) in collaboration with National Institute for Public Health and the Environment (RIVM) and Trimbos-institute, 20xx

66. Zorlu A. Occupational adjustment of immigrants in the Netherlands. Journal of International Migration and Integration. 2013 14(4):711-31.

67. Ravelli, A. C. J. (1999). Prenatal exposure to the Dutch famine and glucose tolerance and obesity at age 50. Universiteit van Amsterdam.

68. Painter RC, de Rooij SR, Bossuyt PM, et al.. Blood pressure response to psychological stressors in adults after prenatal exposure to the Dutch famine. Journal of Hypertension. 2006;24(9):1771–1778. doi: 10.1097/01.hjh.0000242401.45591.e7.

69. Bromly C., Sproston k., Shelton N. (2004) *Scottish Health Survey 2003 summary of findings*, The National Centre for Social Research and Department of Epidemiology and Pubilic Health, University College London https://www.webarchive.org.uk/wayback/archive/20141129163333/http://www.scotland.gov.uk/Publications/2005/12/02160336/03367

Supplementary table 6: Key characteristics of the Simpson's cohort broken down by MRI status, characteristics of the LBC1921 and the wider Scottish population.

	Scottish Population[60,68]	LBC1921[17]	Simpsons		
		Wave 1	All (n=130)	With MRI (n=110)	Without MRI (n=20)
Mean age	-	-	78.4 (30.8)	78.2** (1.4)	79.5** (1.1)
Gender (% female)	51.5	54	90 (69.2)	77 (70)	13 (65)
Adult SES*, mean (SD)	-	2.24 (0.88)	2.6 (0.7)	2.5 (0.8)	2.9 (0.5)
Adult SES, manual, n (%)	-	-	81 (62.3)	64 (58.2)	17 (85)
Current smokers n (%)	31	-	12 (9.2)	8 (7.3)	4 (20)
Hypertension n (%)	41	-	(63)48.5	49** (44.5)	14** (70)
Diabetes n (%)	3.8	-	7 (5.4)	7 (6.4)	0 (0)
Childhood IQ	-	101.6 (14.2)	100.1 (14.8)	101.7 (14.50)	96.0 (15.4)
Low education n (%)	-	-	107 (82.3)	89 (80.9)	18 (90)
Father's SES*, mean (SD)	-	2.73 (0.93)	3.2 (0.8)	3.2 (0.9)	3.25 (0.8)
Father's SES, manual n (%)	-	-	91 (70)	76 (69.1)	15 (75)
Birth weight, (g) mean (SD)	-	-	3331.5 (461.7)	3333.6 (457.2)	3319.65 (497.9)
Ponderal index mean (SD)	-	-	25.7 (4.1)	25.8 (4.2)	25.4 (3.3)

*those with MRI vs those without $p<0.01$; Abbreviations: SES socioeconomic status; Father's SES is based on five categories from professional (1) to unskilled labour; (higher number indicates lower SES); Scottish population figures are given in % only; - is used when data were not available.

Supplementary Table 7: Multiple regression analysis of early life factors and cSVD in STRADL, the Dutch Famine Birth Cohort, the LBC 1936 and the Simpson's cohort.

	STRADL		Dutch Famine		LBC		Simpson's		Meta-analysis	
	OR (95% CI)[a]	p	OR (95% CI)[b]	p	OR (95% CI)[b]	p	OR (95% CI)[b]	p	OR (95% CI)	p
**Moderate/severe cSVD**										
Birth weight	0·94 (0·86-1.04)	0·22	0·95 (0·86-1.05)	0·29	1·001 (0·92-1.10)	0·98	0·94 (0·85-1·04)	0·21	0·96 (0·92-1·01)	0·08
Low education	1·41 (0·41-4·78)	0·59	0·72 (0·27-1.95)	0·52	1·23 (0·31-4·85)	0·77	1·08 (0·34-3·40)	0·90	1·02 (0·57-1·82)	0·95
Manual Father's occupation	1·62 (0·34-7·72)	0·54	0·67 (0·23-1.91)	0·45	0·55 (0·19-1·58)	0·26	0·40 (0·16-1·01)	0·05	0·59 (0·34-1·02)	0·06
**Moderate/severe WMH**										
Birth weight	0·93 (0·83-1·04)	0·21	1·02 (0·93-1·12)	0·68	0·99 (0·90-1·08)	0·79	**0·90 (0·81-0·999)**	**0·048**	0·96 (0·91-1·02)	0·20
Low education	1·12 (0·22-5·82)	0·89	0·53 (0·21-1·37)	0·19	0·81 (0·29-2·84)	0·74	0·88 (0·28-2·7)	0·82	0·74 (0·42-1·28)	0·28
Manual Father's occupation	na	na	1.29 (0·47-3·58)	0·63	1·03 (0·33-3·21)	0·96	0·85 (0·33-2·22)	0·75	1·03 (0·57-1·86)	0·92
**Presence of 1+ lacune**										
Birth weight	0·91 (0·83-1.01)	0·07	0·94 (0·85-1·03)	0·17	0·99 (0·86-1·14)	0·89	0·95 (0·86-1·05)	0·31	**0·94 (0·89-0·99)**	**0·02**
Low education	0·64 (0·13-3·04)	0·57	1·45 (0·53-3·95)	0·47	0·86 (0·09-8·13)	0·89	1·11 (0·34-3·59)	0·86	1·11 (0·57-2·15)	0·76
Manual Father's occupation	0·84 (0·21-3·32)	0·80	0·86 (0·30-2·41)	0·86	0·34 (0·07-1·58)	0·17	0·42 (0·17-1·05)	0·07	0·58 (0·32-1·04)	0·07
**Presence of 1+ CMB**										
Birth weight	0·97 (0·87-1·08)	0·55	0·92 (0·81-1·05)	0·20	1·06 (0·95-1·18)	0·33	1·08 (0·93-1·26)	0·32	1·00 (0·93-1·08)	0·94
Low education	0·69 (0·14-3·30)	0·64	1·05 (0·30-3·74)	0·94	1·06 (0·21-5·45)	0·94	4·07 (0·43-38·82)	0·22	1·12 (0·51-2·47)	0·77
Manual Father's occupation	1·38 (0·28-6·72)	0·69	0·39 (0·11-1·44)	0·16	0·42 (0·13-1·42)	0·17	0·44 (0·12-1·65)	0·22	**0·51 (0·26-0·98)**	**0·04**
**Presence of 1+ infarct**										
Birth weight	0·91 (0·81-1·02)	0·09	0·98 (0·89-1·09)	0·72	0·94 (0·85-1·04)	0·26	0·93 (0·80-1·08)	0·35	**0·94 (0·89-1·00)**	**0·03**
Low education	0·43 (0·05-3·53)	0·43	1·86 (0·62-5·63)	0·27	2·91 (0·35-24·49)	0·33	0·92 (0·17-4·84)	0·92	1·40 (0·64-3·07)	0·40
Manual Father's occupation	0·54 (0·13-2·30)	0·40	1·74 (0·54-5·66)	0·36	0·49 (0·16-1·54)	0·22	0·37 (0·10-1·40)	0·37	0·66 (0·33-1·33)	0·25
**Moderate/severe atrophy**										
Birth weight	0·89 (0·78-1·01)	0·07	1·01 (0·92-1·12)	0·80	0·97 (0·90-1·05)	0·47	1·01 (0·91-1·12)	0·87	0·97 (0·93-1·02)	0·31
Low education	0·71 (0·08-6·25)	0·75	1·46 (0·52-4·12)	0·48	1·35 (0·44-4·12)	0·60	0·51 (0·17-1·48)	0·22	0·99 (0·54-1·80)	0·97
Manual Father's occupation	1·15 (0·12-10·61)	0·90	0·94 (0·32-2·75)	0·91	0·82 (0·31-2·16)	0·82	0·67 (0·26-1·73)	0·41	0·76 (0·48-1·21)	0·25
**Moderate/severe EPVS**										
Birth weight	**0·92 (0·86-0·98)**	**0·01**	0·95 (0·87-1·04)	0·29	0·97 (0·90-1·05)	0·47	0·98 (0·89-1·08)	0·68	**0·95 (0·91-0·99)**	**0·007**
Low education	0·97 (0·42-2·27)	0·94	0·63 (0·24-1.64)	0·34	2·40 (0·68-8·43)	0·17	1.36 (0·45-4·14)	0·59	1.07 (0·65-1.77)	0·80
Manual Father's occupation	0·70 (0·31-1.60)	0·40	0·99 (0·35-2·80)	0·99	0·53 (0·20-1.39)	0·53	0·54 (0·20-1.51)	0·24	0·66 (0·41-1.06)	0·09

cSVD= cerebral small vessel disease; WMH= white matter hyperintensities; CMB= cerebral microbleed; EPVS= enlarged perivascular spaces
NA: due to the low cSVD burden in STRADL participants it was not possible to conduct all analyses.

[a] Analyses adjusted for variables in the table; [b] adjusted for variables in the table and age and sex

Supplementary table 8: Associations between early life factors and whole brain volume as a percentage of the ICV and WMH volume in STRADL, the LBC1936 and Simpson's cohort. All analysis are adjusted for age, sex, hypertension, smoking behaviour, adult SES

	STRADL		Dutch Famine Birth cohort		LBC1936		Simpson's		Meta-analysis	
	B (95% CI)	p	B (95% CI)	p	B (95% CI)	p	B (95% CI)	p	B (95% CI)	p
**Whole brain volume normalised for ICV**										
Ponderal index*	-	-	-	-	0·09 (-0·001-0·001)	0·41	-0·14 (-0·09-0·02)	0·29	-0·04 (-0·18-0·11)	0·39
Birth weight (per 100g)*	0·11 (-0·001-0·004)	0·16	-	-	0·04 (-0·13-0·21)	0·62	-0·20 (-0·39-0·01)	0·40	-0·06 (-0·22-0·10)	0·45
Childhood IQ	-0·06 (-0·002-0·001)	0·34	-	-	0·04 (-0·04-0·12)	0·30	-0·09 (-0·55-0·41)**	0·73	0·04 (-0·04-0·12)	0·31
**WMH volume normalised for ICV**										
Ponderal index*	-	-	0·10 (-0·14-0·59)	0·27	-0·17 (-0·38-0·06)	0·11	-0·11 (-0·30-0·09)	0·19	-0·05 (-0·18-0·09)	0·48
Birth weight (per 100g)*	-	-	0·07 (-0·13-0·27)	0·38	-0·08 (-0·25-0·09)	0·40	-0·08 (-0·27-0·11)	0·43	-0·03 (-0·14-0·07)	0·54
Childhood IQ	-0·03 (-0·07-0·05)	0·44	-	-	-0·07 (-0·15-0·01)	0·12	0·14 (-0·35-0·57)**	0·30	-0·06 (-0·14-0·01)	0·11

* This analysis is also adjusted for gestational age; ** due to sample size this analysis is only adjusted for age and sex; - data were not available

Supplementary figure 2: Forest plot showing associations between features of SVD and ponderal index in the Dutch Famine Birth cohort, LBC 1936 and Simpson's cohort . All analyses are adjusted for age, sex, hypertension, smoking behaviour, adult SES and gestational age. cSVD = total SVD burden

Study or Subgroup	log[Odds Ratio]	SE	Weight	Odds Ratio IV, Random, 95% CI
**1.4.1 Moderate/severe total cSVD**				
Dutch Famine Birth Cohort	-0.0534	0.1141	17.3%	0.95 [0.76, 1.19]
LBC 1936	-0.1625	0.0707	36.3%	0.85 [0.74, 0.98]
Simpson Cohort	-0.0101	0.0584	46.4%	0.99 [0.88, 1.11]
**Subtotal (95% CI)**			**100.0%**	**0.93 [0.84, 1.03]**
Heterogeneity: Tau² = 0.00; Chi² = 2.78, df = 2 (P = 0.25); I² = 28%				
Test for overall effect: Z = 1.42 (P = 0.16)				
**1.4.2 Moderate/severe WMH**				
Dutch Famine Birth Cohort	0.1044	0.1127	19.2%	1.11 [0.89, 1.38]
LBC 1936	-0.0943	0.0657	41.0%	0.91 [0.80, 1.04]
Simpson Cohort	-0.1165	0.0673	39.8%	0.89 [0.78, 1.02]
**Subtotal (95% CI)**			**100.0%**	**0.94 [0.84, 1.04]**
Heterogeneity: Tau² = 0.00; Chi² = 3.00, df = 2 (P = 0.22); I² = 33%				
Test for overall effect: Z = 1.18 (P = 0.24)				
**1.4.3 Presence of 1+ lacune**				
Dutch Famine Birth Cohort	-0.1347	0.1118	20.1%	0.87 [0.70, 1.09]
LBC 1936	-0.1625	0.1369	13.4%	0.85 [0.65, 1.11]
Simpson Cohort	-0.0576	0.0614	66.5%	0.94 [0.84, 1.06]
**Subtotal (95% CI)**			**100.0%**	**0.92 [0.83, 1.01]**
Heterogeneity: Tau² = 0.00; Chi² = 0.72, df = 2 (P = 0.70); I² = 0%				
Test for overall effect: Z = 1.74 (P = 0.08)				
**1.4.4 Presence of 1+ CMBs**				
Dutch Famine Birth Cohort	0.1062	0.1353	19.3%	1.11 [0.85, 1.45]
LBC 1936	0.0198	0.0696	47.9%	1.02 [0.89, 1.17]
Simpson Cohort	0.2207	0.0951	32.8%	1.25 [1.03, 1.50]
**Subtotal (95% CI)**			**100.0%**	**1.11 [0.97, 1.26]**
Heterogeneity: Tau² = 0.00; Chi² = 2.92, df = 2 (P = 0.23); I² = 31%				
Test for overall effect: Z = 1.55 (P = 0.12)				
**1.4.5 Presence of 1+ infarct**				
Dutch Famine Birth Cohort	-0.0101	0.1216	23.9%	0.99 [0.78, 1.26]
LBC 1936	-0.1278	0.0884	45.2%	0.88 [0.74, 1.05]
Simpson Cohort	-0.1054	0.1068	31.0%	0.90 [0.73, 1.11]
**Subtotal (95% CI)**			**100.0%**	**0.91 [0.81, 1.02]**
Heterogeneity: Tau² = 0.00; Chi² = 0.63, df = 2 (P = 0.73); I² = 0%				
Test for overall effect: Z = 1.56 (P = 0.12)				
**1.4.6 Moderate/severe atrophy**				
Dutch Famine Birth Cohort	0.0392	0.1275	9.4%	1.04 [0.81, 1.34]
LBC 1936	0.0392	0.0516	57.5%	1.04 [0.94, 1.15]
Simpson Cohort	-0.0408	0.0681	33.0%	0.96 [0.84, 1.10]
**Subtotal (95% CI)**			**100.0%**	**1.01 [0.94, 1.09]**
Heterogeneity: Tau² = 0.00; Chi² = 0.92, df = 2 (P = 0.63); I² = 0%				
Test for overall effect: Z = 0.33 (P = 0.74)				
**1.4.7 Moderate/severe EPVS**				
Dutch Famine Birth Cohort	-0.102	0.1134	12.4%	0.90 [0.72, 1.13]
LBC 1936	-0.1098	0.0578	47.9%	0.90 [0.80, 1.00]
Simpson Cohort	-0.009	0.0635	39.7%	0.99 [0.88, 1.12]
**Subtotal (95% CI)**			**100.0%**	**0.93 [0.86, 1.01]**
Heterogeneity: Tau² = 0.00; Chi² = 1.48, df = 2 (P = 0.48); I² = 0%				
Test for overall effect: Z = 1.72 (P = 0.09)				

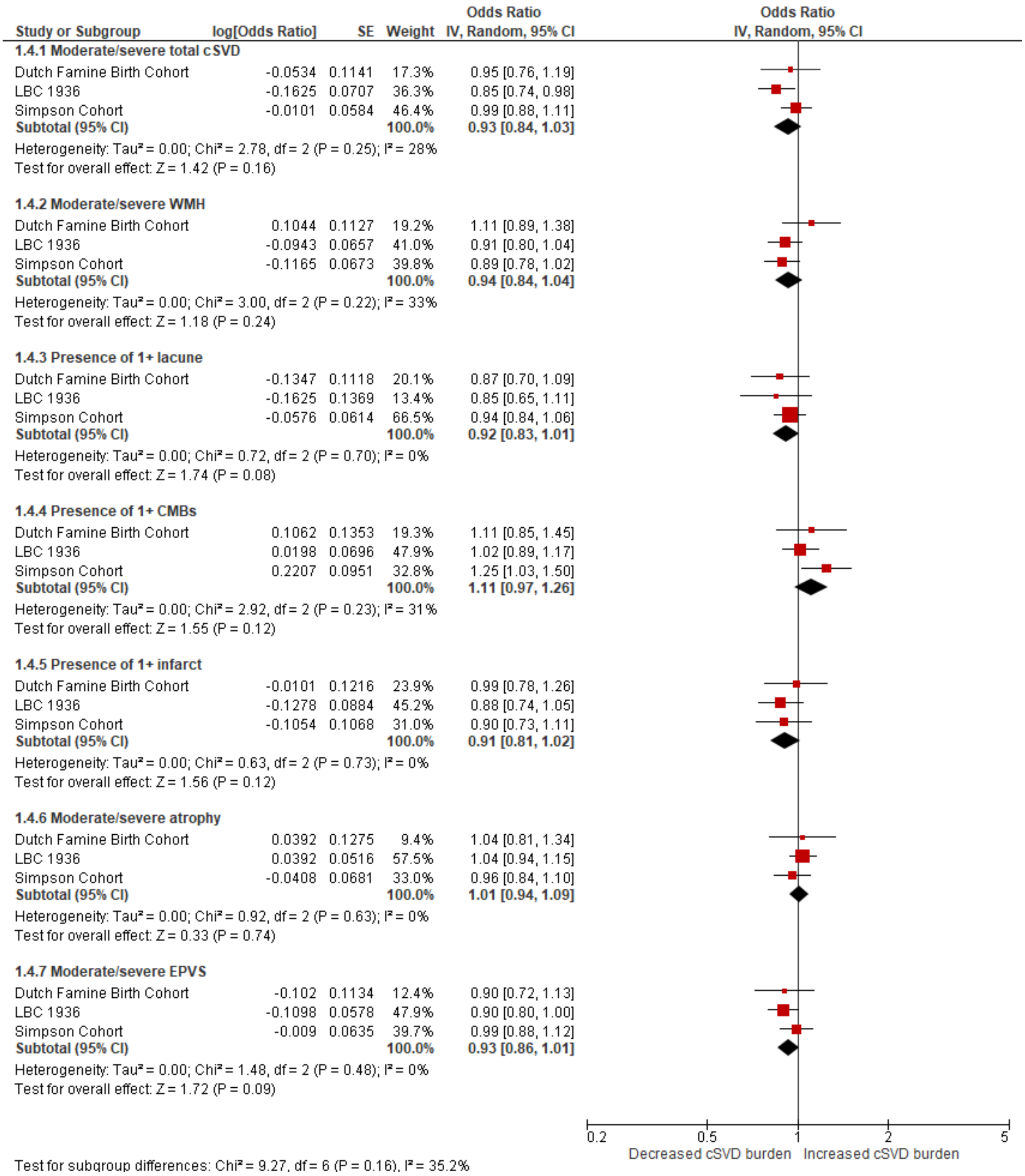

Test for subgroup differences: Chi² = 9.27, df = 6 (P = 0.16), I² = 35.2%

Appendix 2: Results of associations between early life factors and SVD in individual cohorts.

Ponderal index

In the LBC1936, increasing ponderal index was associated with decreased risk of total SVD (per kg/m3 OR=0.85 95%CI=0.74-0.98, Supplementary Fig. 2) independent of age, sex, hypertension, smoking behaviour and adult SES.

Increasing ponderal index was associated with increased risk of cerebral micro-bleeds in the Simpson's cohort but not the expected direction (per kg/m3 OR=1.25 95%CI=1.03-1.50, Supplementary Fig. 2).

Childhood SES

In individual cohorts, low childhood SES was associated with lower total SVD burden (Simpson's cohort only OR=0.36, 95%CI=0.14-0.93) and decreased risk of infarcts (STRADL OR=0.52, 95%CI=0.28-0.94) none of which were in the expected direction (Figure 1D).

Supplementary table 6: Multiple regression analysis of a) Childhood IQ, education, childhood SES and cSVD in STRADL and the LBC 1936

NOTE: It was not possible to include the Simpson's cohort in this analysis due to the small number of participants with childhood IQ scores. Childhood IQ was not available in the Dutch Famine Birth cohort therefore this cohort was not included in this analysis.

All analyses are adjusted for each early life factor in the table and age, sex, hypertension, smoking behaviour and adult SES.

	STRADL		LBC 1936		Meta-analysis	
	OR (95% CI)	p	OR (95% CI)	p	OR (95% CI)	p
**Moderate/severe cSVD**						
Childhood IQ	0·97 (0·93-1.004)	0·06	0·99 (0·97-1.00)	0·07	**0·98 (0·97-0·997)**	**0·02**
Low education	0·87 (0·42-1.80)	0·71	0·99 (0·56-1.75)	0·97	0·94 (0·60-1.48)	0·80
Manual Father's occupation	1.11 (0·54-2·28)	0·78	1.32 (0·75-2·32)	0·34	1.24 (0·79-1.93)	0·35
**Moderate/severe WMH****						
Childhood IQ*	0·98 (0·94-1.02)	0·34	0·99 (0·98-1.01)	0·29	0·99 (0·98-1.00)	0·20
Low education	1.19 (0·46-3·06)	0·72	0·79 (0·47-1.33)	0·37	0·87 (0·55-1.37)	0·54
Manual Father's occupation	1.28 (0·51-3·21)	0·60	1.25 (0·75-2·08)	0·39	1.26 (0·80-1.96)	0·32
**Presence of 1+ lacune**						
Childhood IQ*	1.02 (0·95-1.09)	0·66	0·97 (0·95-1.00)	0·04	0·98 (0·95-1.02)	0·29
Low education	0·34 (0·10-1.16)	0·08	1.10 (0·38-3·18)	0·86	0·64 (0·20-2·01)	0·44
Manual Father's occupation	2·74 (0·79-9·50)	0·11	**0·30 (0·13-0·71)**	**0·01**	0·87 (0·10-7·48)	0·90
**Presence of 1+ CMB**						
Childhood IQ*	**0·95 (0·90-0·995)**	**0·03**	0·995 (0·98-1.01)	0·59	0·98 (0·93-1.02)	0·32
Low education	1.06 (0·42-2·67)	0·91	1.35 (0·67-2·71)	0·41	1.24 (0·71-2·18)	0·45
Manual Father's occupation	0·78 (0·33-1.87)	0·58	1.20 (0·62-2·31)	0·58	1.02 (0·61-1.73)	0·93
**Presence of 1+ infarct**						
Childhood IQ*	0·98 (0·89-1.08)	0·68	**0·98 (0·97-1.00)**	**0·03**	**0·98 (0·97-0·997)**	**0·02**
Low education	1.14 (0·20-6·50)	0·88	1.07 (0·56-2·05)	0·84	0·95 (0·60-1.48)	0·80
Manual Father's occupation	0·67 (0·13-3·45)	0·63	0·77 (0·43-1.38)	0·38	1.24 (0·79-1.93)	0·35
**Moderate/severe atrophy**						
Childhood IQ*	1.04 (0·98-1.10)	0·24	0·98 (0·99-1.01)	0·67	1.01 (0·96-1.05)	0·79
Low education	0·60 (0·19-1.87)	0·37	0·89 (0·57-1.35)	0·58	0·82 (0·55-1.23)	0·34

Manual Father's occupation	2·12 (0·67-6·68)	0·20	0·88 (0·59-1.31)	0·51	1.13 (0·48-2·64)	0·78
**Moderate/severe EPVS**						
Childhood IQ*	0·98 (0·94-1.02)	0·37	1.01 (0·99-1.02)	0·48	1.00 (0·98-1.02)	0·85
Low education	1.29 (0·58-2·90)	0·53	0·91 (0·59-1.38)	0·65	0·99 (0·67-1.44)	0·94
Manual Father's occupation	0·82 (0·38-1.78)	0·62	1.04 (0·69-1.55)	0·86	0·99 (0·69-1.42)	0·94

* OR is given per 1 point increase in IQ; moderate/severe WMH= Fazekas deep WMH score ≥2 or periventricular WMH score

Supplementary figure 3: Scatterplots of association between age 11 IQ and a) WMH volume b) whole brain volume in the LBC 1936 and Simpson's cohort.

a)

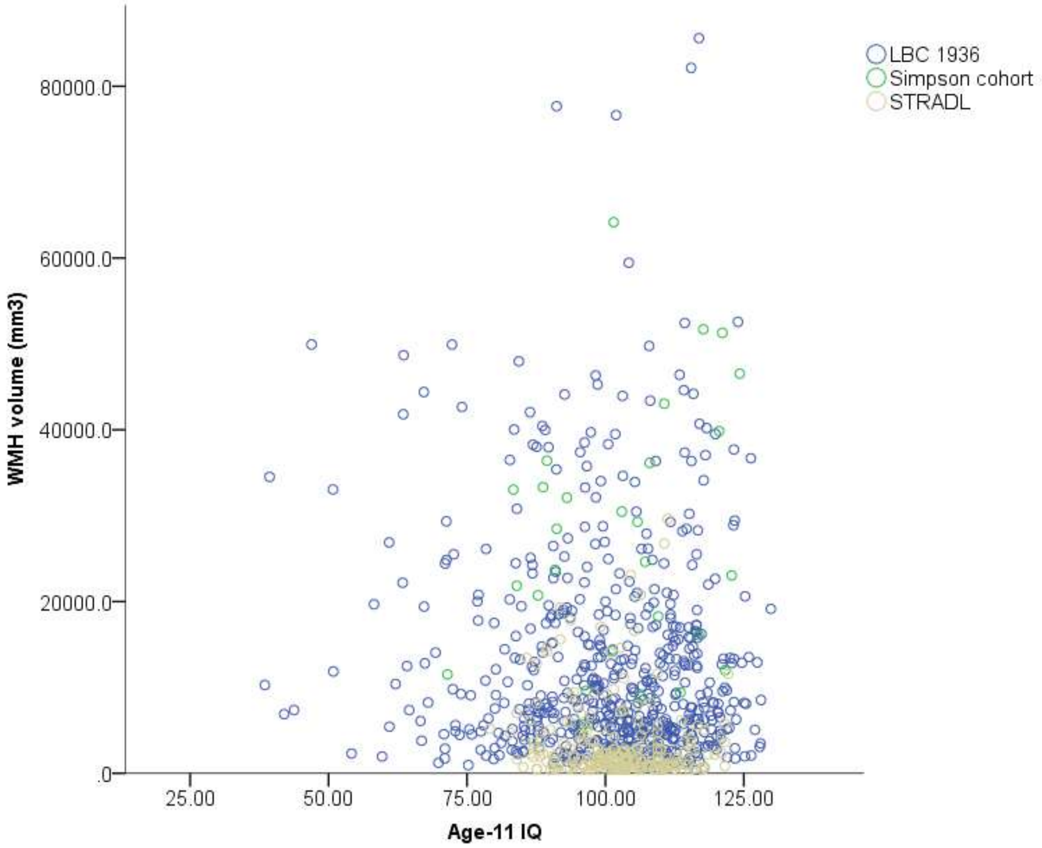

b)

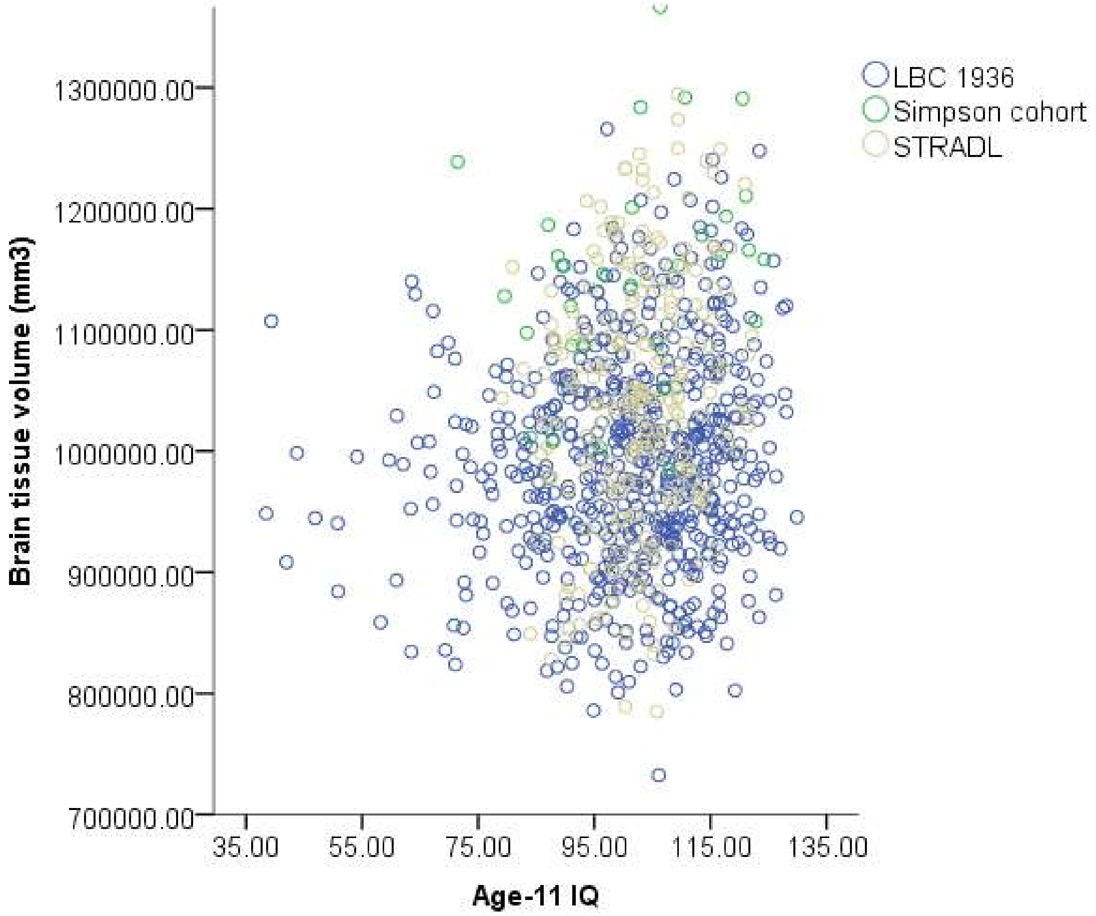

LBC 1936
Simpson cohort
STRADL
1300000.00
1200000.00
1100000.00
1000000.00
900000.00
800000.00
700000.00
Brain tissue volume (mm3)
35.00
55.00
75.00
95.00
115.00
135.00
Age-11 IQ

Supplementary figure 4: Forest plots showing mean white matter hyperintensity (WMH) volume for those with a) low vs high levels of education b) low vs high childhood SES, random effects model for the mean difference.

a)

	Low education			High education				Mean Difference
**Study or Subgroup**	**Mean**	**SD**	**Total**	**Mean**	**SD**	**Total**	**Weight**	**IV, Random, 95% CI**
STRADL	369.3054	446.2846	112	284.6279	488.4692	358	71.3%	84.68 [-12.23, 181.59]
Dutch Famine Birth Cohort	404.32466	430.843009	73	476.86977	625.637723	43	15.0%	-72.55 [-284.05, 138.96]
LBC 1936	1,186.1671	1,129.7139	432	1,234.3786	1,297.4641	168	13.4%	-48.21 [-271.46, 175.04]
Simpson Cohort	3,101.1835	2,586.057	87	3,193.9707	3,017.6053	20	0.3%	-92.79 [-1522.58, 1337.00]
**Total (95% CI)**			**704**			**589**	**100.0%**	**42.72 [-39.10, 124.54]**

Heterogeneity: Tau² = 0.00; Chi² = 2.53, df = 3 (P = 0.47); I² = 0%
Test for overall effect: Z = 1.02 (P = 0.31)

Mean Difference
IV, Random, 95% CI

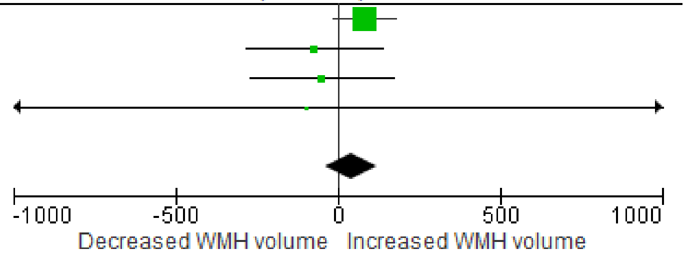

Negative mean difference= low education (<11 years) decreases WMH volume; positive difference= low education increases WMH volume.

b)

	Low SES			High SES				Mean Difference
**Study or Subgroup**	**Mean**	**SD**	**Total**	**Mean**	**SD**	**Total**	**Weight**	**IV, Random, 95% CI**
STRADL	320.486	433.0174	308	249.6962	446.8204	157	64.4%	70.79 [-14.20, 155.78]
Dutch Famine Birth Cohort	433.295	440.784095	62	523.97813	446.8204	32	19.5%	-90.68 [-280.43, 99.07]
LBC 1936	1,217.5348	1,185.047	152	1,147.0013	1,159.965	448	15.4%	70.53 [-146.33, 287.39]
Simpson Cohort	2,951.0752	2,592.6549	74	3,494.0246	2,799.9478	33	0.6%	-542.95 [-1666.14, 580.24]
**Total (95% CI)**			**596**			**670**	**100.0%**	**35.31 [-54.49, 125.12]**

Heterogeneity: Tau² = 1378.52; Chi² = 3.42, df = 3 (P = 0.33); I² = 12%
Test for overall effect: Z = 0.77 (P = 0.44)

Mean Difference
IV, Random, 95% CI

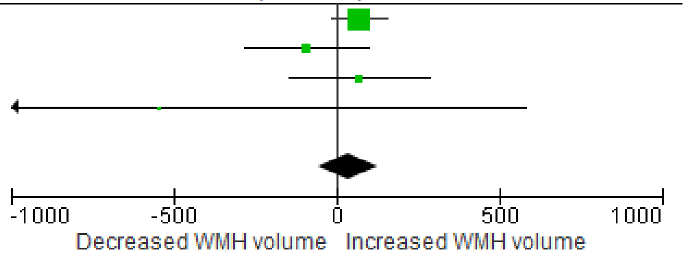

Negative mean difference= low SES (manual paternal occupation) decreases WMH volume; positive difference= low SES increases WMH volume.

Supplementary figure 5: Forest plots showing total brain volume for those with a) low vs high levels of education b) low vs high childhood SES, random effects model for the mean difference.

a)

	Low education			High education				Mean Difference
Study or Subgroup	Mean	SD	Total	Mean	SD	Total	Weight	IV, Random, 95% CI
STRADL	10,617.6675	1,022.9806	248	10,728.7594	1,110.6724	798	47.1%	-111.09 [-259.91, 37.73]
LBC 1936	9,870.8599	889.4543	432	10,051.481	869.1933	168	45.0%	-180.62 [-336.54, -24.70]
Simpson's Cohort	11,250.4818	949.4492	87	11,824.3877	1,040.7666	20	7.9%	-573.91 [-1071.76, -76.05]
**Total (95% CI)**			**767**			**986**	**100.0%**	**-178.86 [-325.07, -32.66]**

Heterogeneity: Tau² = 6040.58; Chi² = 3.13, df = 2 (P = 0.21); I² = 36%
Test for overall effect: Z = 2.40 (P = 0.02)

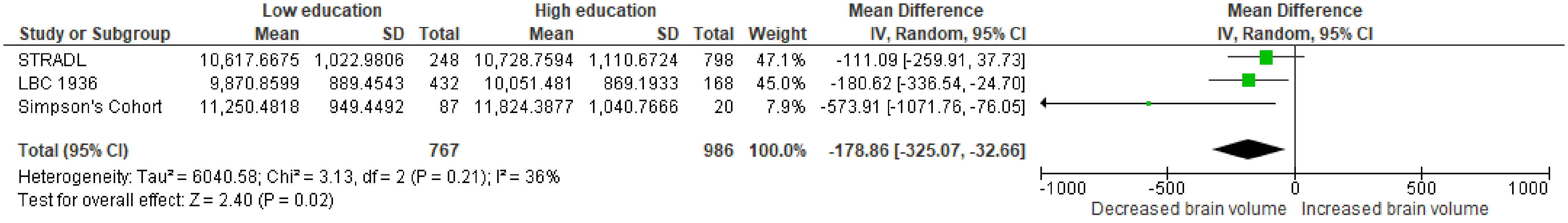

Negative mean difference= low education (<11 years) decreases brain volume volume; positive difference= low education increases brain volume

b)

	Low SES			High SES				Mean Difference
Study or Subgroup	Mean	SD	Total	Mean	SD	Total	Weight	IV, Random, 95% CI
STRADL	10,600.4456	1,090.3803	701	10,909	1,064.2719	345	52.1%	-308.55 [-446.86, -170.25]
LBC 1936	9,904.5153	899.5036	152	9,971.299	849.2843	448	47.9%	-66.78 [-229.98, 96.41]
Simpson's Cohort	11,307.463	96,551.9905	74	11,471.462	1,042.7152	33	0.0%	-164.00 [-22165.40, 21837.40]
**Total (95% CI)**			**927**			**826**	**100.0%**	**-192.74 [-403.96, 18.47]**

Heterogeneity: Tau² = 17313.15; Chi² = 4.91, df = 2 (P = 0.09); I² = 59%
Test for overall effect: Z = 1.79 (P = 0.07)

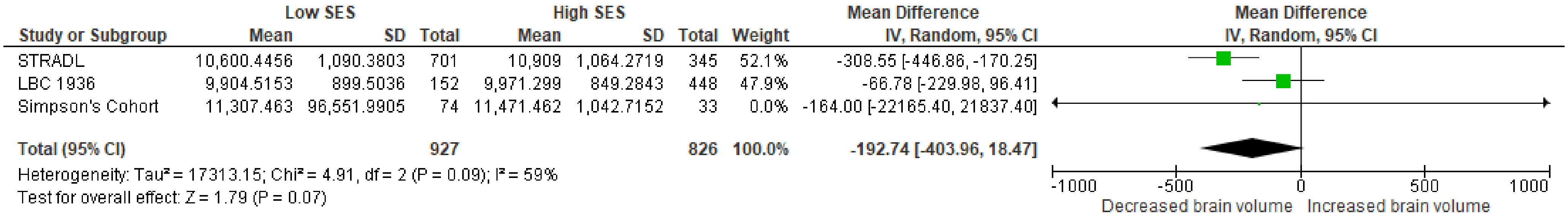

Negative mean difference= low SES (manual paternal occupation) decreases brain volume; positive difference= low SES increases brain volume.

Supplementary table 7: Associations between early life factors and whole brain volume as a percentage of the ICV and WMH volume in STRADL, the LBC 1936 and Simpson's cohort. All analysis are adjusted for age, sex, hypertension, smoking behaviour, adult SES.

	STRADL		Dutch Famine Birth cohort		LBC 1936		Simpsons		Meta-analysis	
	B (95% CI)	p	B (95% CI)	p	B (95% CI)	p	B (95% CI)	p	B (95% CI)	p
**Whole brain volume as a % of ICV**										
Low vs high education level	0·01 (-0·01-0·02)	0·76	-	-	0·03 (-0·05-0·11)	0·46	-0·14 (-0·06-0·33)	0·22	0·01(-0·04-0·06)	0·75
Low vs high childhood SES	-0·01 (-0·02-0·01)	0·81	-	-	0·04 (-0·04-0·12)	0·14	-0·09 (-0·29-0·11)	0·40	0·01(-0·05-0·07)	0·74
**WMH volume**										
Low vs high education level	0·06 (0·00-0·001)	0·21	0·08 (-0·12-0·28)	0·38	0·004 (-0·07-0·08)	0·90	-0·01 (-0·21-0·19)	0·97	0·02 (-0·03-0·08)	0·33
Low vs high childhood SES	-0·02 (-0·001-0·001)	0·61	0·16 (-0·10-0·50)	0·28	0·01 (-0·07-0·09)	0·85	-0·17 (-0·36-0·03)	0·08	-0·01 (-0·06-0·51)	0·87
